# Wing Interferential Patterns (WIPs) and machine learning, a step toward automatized tsetse (*Glossina *spp.) identification

**DOI:** 10.1038/s41598-022-24522-w

**Published:** 2022-11-22

**Authors:** Arnaud Cannet, Camille Simon-Chane, Mohammad Akhoundi, Aymeric Histace, Olivier Romain, Marc Souchaud, Pierre Jacob, Pascal Delaunay, Darian Sereno, Philippe Bousses, Pascal Grebaut, Anne Geiger, Chantel de Beer, Dramane Kaba, Denis Sereno

**Affiliations:** 1 Direction des affaires sanitaires et sociales de la Nouvelle-Calédonie, Nouméa, New Caledonia France; 2 ETIS UMR 8051, Cergy Paris University, ENSEA, CNRS, 95000 Cergy, France; 3 Parasitology-Mycology, Hôpital Avicenne, AP-HP, Bobigny, France; 4 Inserm U1065, Centre Méditerranéen de Médecine Moléculaire (C3M), Université de Nice-Sophia Antipolis, Nice, France; 5 Parasitologie-Mycologie, Hôpital de L’Archet, Centre Hospitalier Universitaire de Nice, (CHU), Nice, France; 6 InterTryp, Univ Montpellier, IRD-CIRAD, Parasitology Infectiology and Public Health Research Group, Montpellier, France; 7 MIVEGEC, Univ Montpellier, CNRS, IRD, Montpellier, France; 8 Insect Pest Control Laboratory, Joint FAO/IAEA Center of Nuclear Techniques in Food and Agriculture, Vienna, Austria; 9 Epidemiology, Parasites & Vectors, Agricultural Research Council - Onderstepoort Veterinary Research (ARC-OVR), Onderstepoort, South Africa; 10 Institut Pierre Richet, Institut National de Santé Publique, Abidjian, Côte d’Ivoire

**Keywords:** Parasitology, Epidemiology, Optical imaging, Taxonomy, Entomology

## Abstract

A simple method for accurately identifying *Glossina spp* in the field is a challenge to sustain the future elimination of Human African Trypanosomiasis (HAT) as a public health scourge, as well as for the sustainable management of African Animal Trypanosomiasis (AAT). Current methods for Glossina species identification heavily rely on a few well-trained experts. Methodologies that rely on molecular methodologies like DNA barcoding or mass spectrometry protein profiling (MALDI TOFF) haven’t been thoroughly investigated for *Glossina *sp. Nevertheless, because they are destructive, costly, time-consuming, and expensive in infrastructure and materials, they might not be well adapted for the survey of arthropod vectors involved in the transmission of pathogens responsible for Neglected Tropical Diseases, like HAT. This study demonstrates a new type of methodology to classify *Glossina* species. In conjunction with a deep learning architecture, a database of Wing Interference Patterns (WIPs) representative of the *Glossina* species involved in the transmission of HAT and AAT was used. This database has 1766 pictures representing 23 *Glossina* species. This cost-effective methodology, which requires mounting wings on slides and using a commercially available microscope, demonstrates that WIPs are an excellent medium to automatically recognize Glossina species with very high accuracy.

## Introduction

*Glossina* (*G.*) spp. (Diptera: Glossinidae), also called tsetse flies, are well-known cyclical vectors of Human African Trypanosomiasis (HAT) that cause sleeping, also known as sleeping sickness in humans, and African Animal Trypanosomiasis (AAT) or nagana in domestic livestock. This unique genus is divided into three monophyletic subgenera, which are *Glossina* (Morsitans group), *Nemorhina* (Palpalis group), and *Austenina* (Fusca group). Besides fossil species of *Glossina* flies, including *G. oligocena* (scudder, 1892), *G. sedialensis* (Miller, 1892), *G. lineolata* (Rowley, 1908), *G. veterna* (cockerell, 1916), and *G. osborni* (Cockerell, 1908)^1^, there are currently 31 named tsetse species and subspecies^2^. Some of these species were subdivided into multiple subspecies because of minor but constant morphological differences, in particular, the genitalia structures of males and females. Because of minor but constant anatomical differences, in particular the genitalia structures of males and females, they are divided into subspecies. Among the 31 currently named Glossina spp, 17 are suspected or proven vectors of Trypanosoma parasites; these are *Glossina palpalis palpalis* (Robineau-Desvoidy, 1830), *Glossina palpalis gambiensis* (Vanderplank, 1949), *Glossina fuscipes fuscipes* (Newstead, 1910), *Glossina fuscipes quazensis* (Pires, 1948), *Glossina tachninoides* (Westwood, 1850), *Glossina fuscipes martinii* (Zumpt, 1935) and *Glossina caliginea* (Austen, 1911), *Glossina swynertoni* (Austen, 1923), *Glossina morsitans morsitans* (Westwood, 1850), *Glossina morsitans submorsitans* (Newstead 1910), *Glossina morsitans centralis* (Machado, 1970)*, Glossina pallidipides* (Austen, 1903), and *Glossina longipalpis* (Wiedman, 1830), *G. austeni* (Newstead, 1912), *G. pallicera pallicera* (Bigot, 1891), *G. longipenis* (Corti, 1895), *G. brevipalpis* (Newstead, 1910). They are proven, or suspected vectors of either *Trypanosoma brucei gambiense* Dutton 1902 or *Trypanosoma rhodesiensis* Stephens and Fanntham 1910 parasites responsible for the HAT and are also involved in the cyclic transmission of trypanosomes accountable for the AAT like *Trypanosoma vivax* Zienman, 1905, *Trypanosoma congolensis* Brodenn, 1904*, Trypanosoma brucei brucei* Plimmer and Bradford, 1899, and *Trypanosoma simiae* Bruce 1895^3–5^. The Democratic Republic of the Congo has the highest species and subspecies richness (16 *Glossina spp* recorded). The highest richness of *Glossina spp* involved in the transmission of parasites responsible for AAT or HAT is recorded in 7 countries, Senegal, Nigeria, The Republic Democratic of Congo, The Central African Republic, Sudan, and Ethiopia^6^. To get insight into the trypanosomiasis prevalence risk, taking information concerning the tsetse population is needed, particularly regarding the species composition, relative abundance, and sex composition. Although sex determination in *Glossina* spp is easy to perform, species differentiation requires in-depth knowledge of morphological criteria, including color, size, and anatomical features such as antennae in male and female genitalia^2^. In the 1990s, software-assisted taxonomy of *Glossina* spp was developed^7^ allowing species identification. In addition, protein profiling using matrix-assisted laser desorption/ionization time-of-flight mass spectrometry (MALDI-TOF–MS) developed during the last decade as a tool for identifying and phylogenetic classification of microorganisms^8^ and has been subsequently applied to arthropod vectors, including *Glossina *spp.^9–16^.

Since the 2010s, WIPs (Wing Interference Patterns) have received significant attention for their potential as a diagnostic method for species identification, used in taxonomic and systematic studies^17–19^. The transparent wings with a thin membrane*, i.e*., mainly in small insects, allow the formation of a colored pattern via thin-film interference. In a dark and light-absorbing environment with incoming external light (sunshine, for example), conspicuous WIPs are displayed on the wing membranes. These WIPs significantly vary among specimens belonging to different species but moderately between specimens for the same species or between sexes. The observed newton color series is similar to that appearing on a soap bubble and is directly proportional to the thickness of the wing membrane at any given point. Unlike the angle-dependent iridescence effect of a flat film, wing structures in an insect’s thin wing membrane act as diopters ensuring the WIPs appear essentially non-iridescent^18^. The role played by WIPs on sexual selection in *Drosophila melanogaster* was addressed, demonstrating that males with more vivid wings are more attractive to females than males with dull wings. These experimental results add a visual element to the mating array of Drosophilia^20^. Even if WIPs are helpful optical characters that can help solve some taxonomic problems, their use for dipteran insect identification has not been thoroughly investigated. These characteristics carried on wings are shared by a wide array of small insects with hyaline wings, including arthropods of veterinary and medical importance. Therefore, combining WIPs imaging with up-to-date image classification methodologies could pave the way to robust and reproducible identification of species and subspecies of tsetse for medical entomology purposes.

Deep learning (DL) is a branch of machine learning (ML) and artificial intelligence (AI). It is a core technology of today's Fourth Industrial Revolution (4IR or Industry 4.0). Due to its learning capabilities from data, DL technology originated from an artificial neural network (ANN) and is widely applied in various application areas. However, building an appropriate DL model is *challenging* due to the dynamic nature and variations in real-world problems and data. Hence, as a proof of concept, we investigate the reliability and specificity of WIPs for species diagnostic in *Glossina *spp. We first examined the feasibility of detecting WIPs on the wings of a relatively large (6 to more than 10 mm) tsetse. Then we validated the stability of the recorded pattern according to various parameters: light polarization, wing orientation (intrados, extrado left and right), the insect genera, and geographical origin. Since field-collected tsetse are stored in ethanol, another goal has been to investigate if such a conservative process significantly influences the color pattern display on wings. We then build up a reference database representative of 23 species or subspecies and combine WIPs and DL approaches to train a classifier to classify WIPs pictures taken from the wings of *Glossina *spp.

## Material and methods

### Tsetse selection and storage

To establish the first tsetse reference collection of WIPs, we use well-established laboratory-reared tsetse flies listed in Table 1. In addition, we selected tsetse samples of ARIM collection belonging to IRD (Institut de Recherche pour le Développement) (https://arim.ird.fr/). Finally, we included field-caught tsetse specimens as well. The description of the samples used in this study is given in Table 1.

**Table 1:** List of named Glossina species and subspecies and description of samples included in the dataset.

	Origin	Sampling year	nb	Identification
***Glossina * spp.** **in the database**				
*G. austeni* (Newstead, 1912)	NK	1950	**4**	F. Pias
*G. brevipalpis* (Newstead, 1910)	Kenya	1978	**13**	AF. Snow
*G. calliginea* (Austen, 1911)	Cameroun	1951	**23**	JP. Adam
*G. fusca fusca* (Walker, 1849)	Ivory Coast	1951	**20**	J. Brunhes
*G. fuscipes fuscipes* (Newstead 1911)	Colony, NK	2013, 1956	**301**	B. Tchikaya, J. Rageau
*G. fuscipes quazensis* (Pires, 1948)	Cameroun, RDC	2014, 1967	**96**	P. Grebault, J. Brunhes
*G. haningtoni* (Newstead& Evans, 1922)	Cameroun	1951	**1**	J. Rageau
*G. longipalpis* (Wiedmann, 1830)	Ivory Coast, Congo	1957	**30**	A. Rickenbach, JP. Adam
*G. longipenis* (Corti, 1895)	NK	NK	**4**	J. Brunhes
*G. medicorum* (Austen, 1911)	NK	1956	**12**	A. Richenback
*G. morsitans centralis* (Machado, 1970)	Burundi	1971	**22**	J. Brunhes
*G. morsitans morsitans* (Westwood, 1850)	Colony, Ethiopia	1955, 1969, 2014	**146**	B. Tchicaya, J. Brunhes, M Ovazza
*G. morsitans submorsitans* (Newstead, 1910)	Cameroun, Senegal	1969, 1971	**30**	J. Brunhes
*G. nashi* (Potts, 1951)	RCA	1993	**2**	JP. Gouteux
*G. nigrofusca nigrofusca* (Newstead, 1910)	Ivorycoast	1977	**11**	J. Brunhes
*G. pallicera pallicera* (Bigot, 1891)	Ivory coast	1978	**14**	A. Challier
*G. pallidipes* (Austen, 1903)	Kenya, Ethiopia, Zimbabwe	1951, 1956	**117**	A. Rickenback
*G. palpalis gambiensis* (Vanderplank, 1949)	Colony, Ivory coast, Burkina Faso, Mali	1945, 1960, 2014	**195**	B. Tchicaya, G Le Goff, D Kaba
*G. palpalis palpalis* (Robineau-Desvoidy, 1830)	Colony, Ivory coast	2013, 2014	**620**	B. Tchicaya, D. Kaba
*G. swynertoni* (Austen, 1923)	Kenya	1993	**3**	JP. Hervy
*G. tabaniformis* (Westwood, 1850)	Cameroun, Congo	1953	**2**	J. Rageau
*G. tachinoïdes* (Westwood, 1850)	Colony, Burkina Faso	1962, 1969, 2014	**94**	B.Tchicaya, J. Brunhes, JP Adam
*G. vanhoofi* (Henrard, 1952)	NK	NK	2	J. Brunhes
***Glossina* spp. not in the database**				
*G. fleuscipleuris* (Austen, 1911)				
*G. frezili* (Gouteux, 1988)				
*G. fusca congolensis* (Newstead& Evans, 1921)				
*G. fuscipes martinii* (Zumpt, 1935)				
*G. nigrofusca hopkinsi* (Van Emden, 1944)				
*G. schwetzi* (Newstead & Evans, 1921)				
*G. severini* (Newstead, 1913)				
*G. pallicera newsteadi* (Austen, 1929)				

NK, Not know, RDC, Republic Democratic of the Congo. For species not included in the database, this can be due to a lack of available specimens or a lack of well-preserved specimens to take reliable pictures of WIPs.

### Image acquisition and database construction

Insect wings were dissected and deposited on a glass slide. For samples preserved in 70° ethanol, wings were layered overnight at room temperature on a glass slide before being photographed. For image acquisition, a cover slide is deposited on the sample. The picture was taken using a Keyence™ VHX 1000 microscope, the VH-Z20r camera, and a Keyence VH K20 adapter allowing an illumination incidence of 10°. Image acquisition was performed using the High Dynamic Range (HDR) function. To exclude the size as a discriminating parameter of *Glossina* identification, magnification was adjusted to ensure constant-size pictures.

The numerical parameter settled were as follow:

- Camera White Balance: 3200 K
- Shutter Speed: preset 1/15(sec)
- Gain: 0 dB
- Frame rate 15F/s

HDR function:

- Brightness: 15%
- Texture: 15%
- Contrast: 45%
- Color: 100%

**Figure 1:**
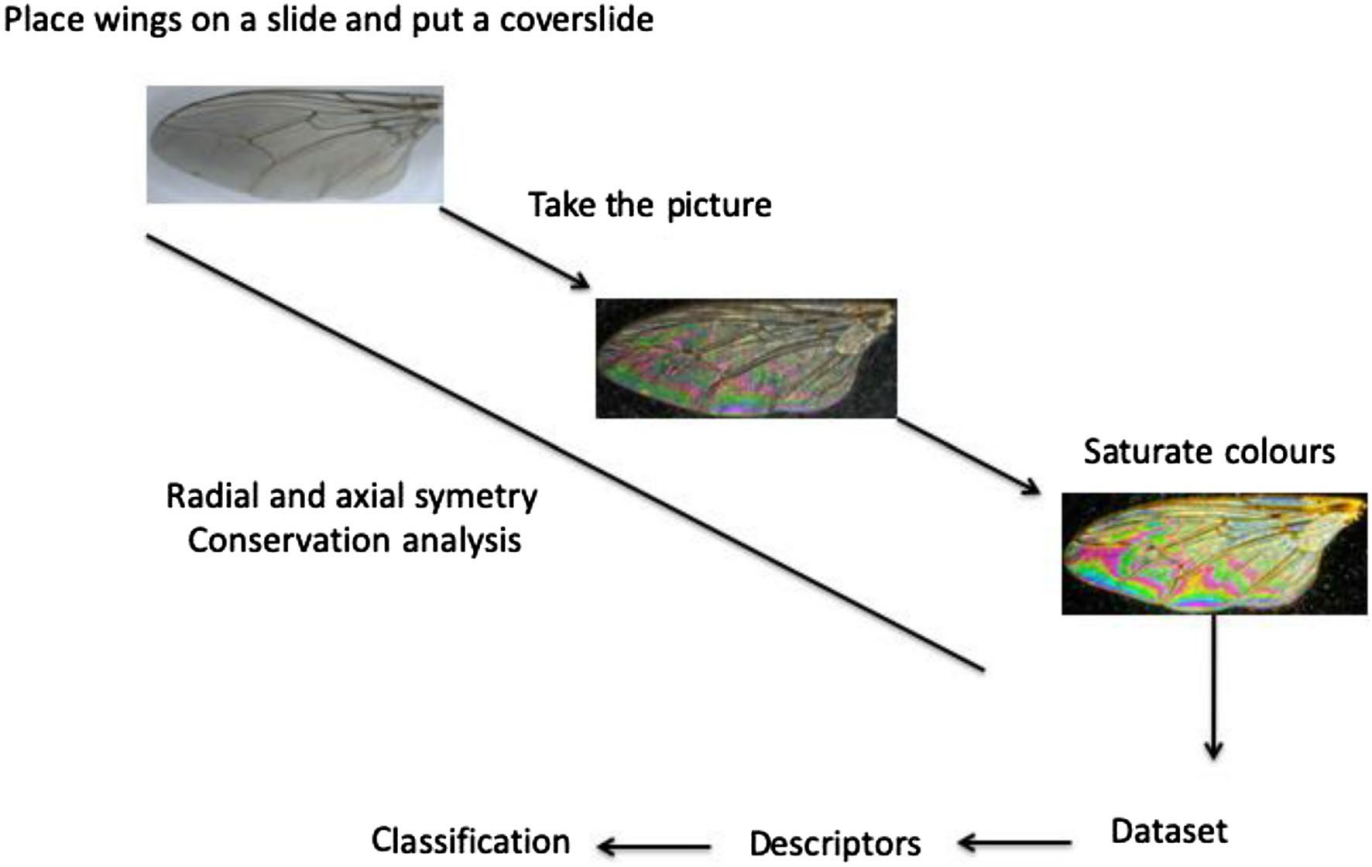
Schematic figure of the flowchart of imaging, labeling process and inclusion in the dataset.

Next, the luminosity, contrast, shadow, reflection, and saturation were settled up at 80, 100, 0, 0, and 100%, respectively, using window 7 familial edition; see Fig. 1 for the flow chart.

### Drawing pictorial identification key using WIPs pictures

The color patterns (blue, red, yellow) were drawn from enlarged image layers of the WIPs. The layers were scanned, and colors were added using the "top color filling" option of the free software GIMP version 2.10.30 (Available from: https://www.gimp.org)^21^.

### Image pre-processing, WIPs dataset splitting for training and testing

**Figure 2:**
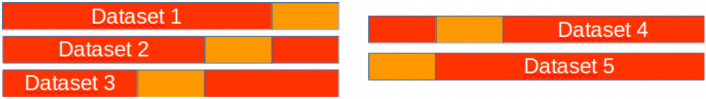
Schematic representation of the dataset splitting for learning (red) and testing (orange).

**Figure 3:**
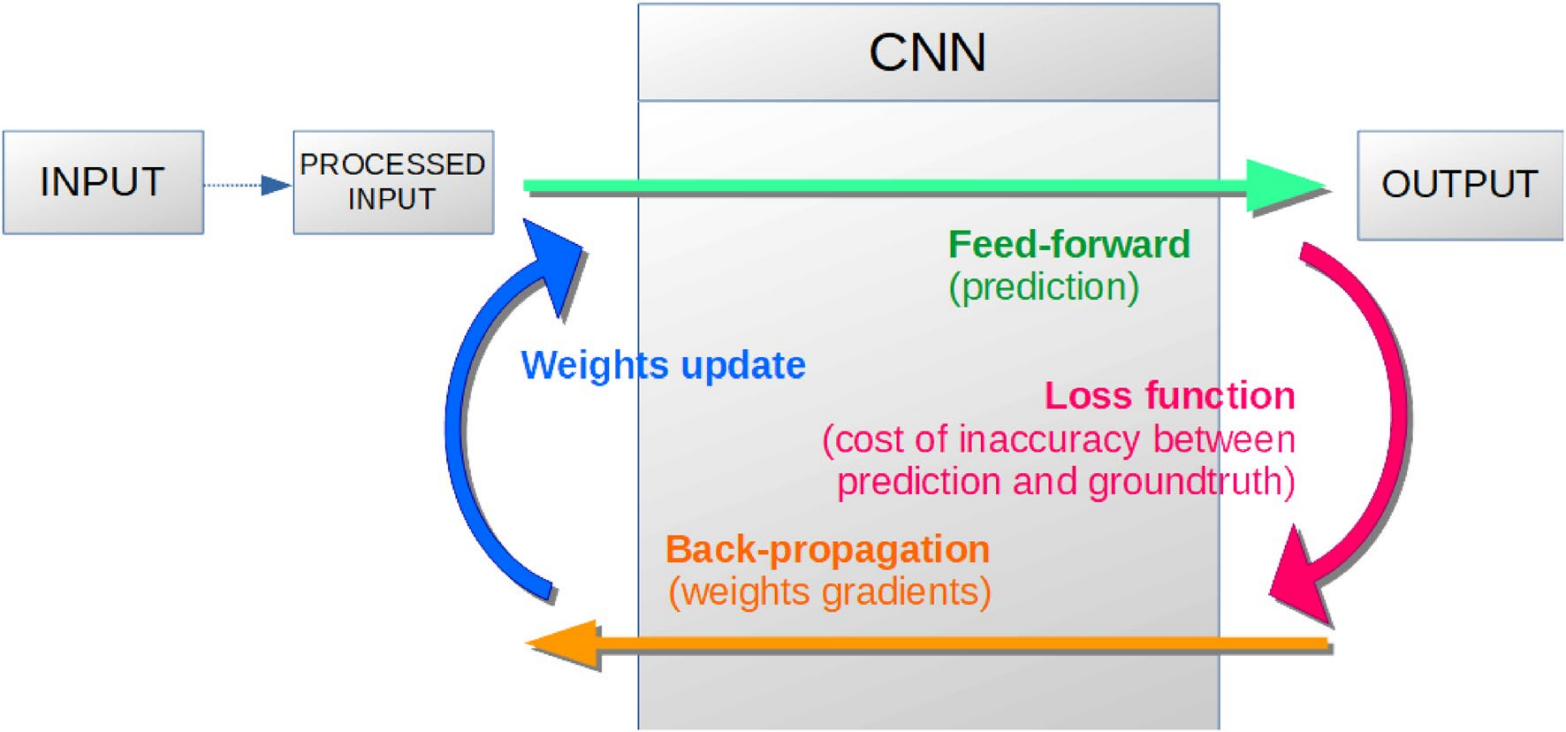
Schematic representation of the pipeline process developed for Glossina identification using the Convolutional Neural Network (CNN) approach.

The WIPS dataset, which consisted of 5516 pictures of dipterann insects WIPs, including 1766 pictures of Glossina, is available on request for glossina images. The link is provided in the Additionnal information section Specimens genus was not considered to build the training and test datasets. All non-*Glossina* images were discarded from this publicly available dataset. Under-sampled species or subspecies (less than 10 samples) are also discarded to prevent overfitting. Images were resized to 256 and 116 pixels for width and height, respectively. Pixel values were normalized within the (0, 1) range^22^. The dataset was then prepared for k-fold cross-validation, with k = 5 (Fig. 2). The dataset was randomly shuffled and partitioned into k equal-size subsets with similar class distributions. A separate model was evaluated for each subgroup using this subset for validation and the remaining k − 1 as training data (Fig. 3). This strategy allowed measuring the mean accuracy of 5 distinct models and was the most accurate neural network performance estimation method.

### Training the neural network

To automatically classify tsetse species and subspecies with the aforementioned dataset, the original MobileNet^23^, ResNet^24^, and YOLOv2^25^ architecture were considered. To consider the smaller size of the database as compared to classic DL databases like Imagenet, a smaller architecture was developed for image recognition and classification. The first architecture inspired from MobileNet takes advantage of depth-wise convolution^23^. Our architecture uses one less scale (the scale with 1024 filters was not used) and one depth-wise convolution per scale, compared to MobileNet, which had 2 depth-wise convolutions. We used batch normalization^26^ to speed up and stabilize training.

Contrary to MobileNet, we use two fully connected layers, similarly to VGG^27^. For YOLOv2, we reproduced the architecture of DarNet-19^25^. As the entire architecture tends to overfit the training set (see Fig. 1), we examined two reduced architectures, i.e., using 1 or 2 scales less than the original network. We called them DarkNet-9 (8 convolution layers and 1 classification layer) and DarkNet-14 (13 convolution layers and 1 classification layer). Finally, we reproduced the ResNet18 architecture from^24^ and trained it from random initialization. Even if this architecture seems too deep for this task compared to our other architectures, residual connections allow convergence of the training procedure and excellent results. We also tested a more standard approach based on extracting SURF descriptors, a Bag of Features (BoF) representation using a 4000 codewords dictionary, and an SVM with a polynomial kernel. This was a similar approach to Sereno et al.^22^: they used SIFT with VLAT representations and a linear SVM as a classifier. VLAT, which was a second-order statistic-based method, has much more information than the zero-order BoF one but has the advantage of being smaller and faster.

From the implementation perspective, all images were resized to 256 × 256 pixels. During the training process, various image augmentation techniques were applied, such as horizontal and vertical flipping, random rotation of the image, zooming into the picture, etc. The main classic idea here was to strengthen the existing WIPs dataset through random variations of images. A pipeline overview of the complete training procedure is shown in Fig. 3.

### Test for identification process following image transformation

**Figure 4:**
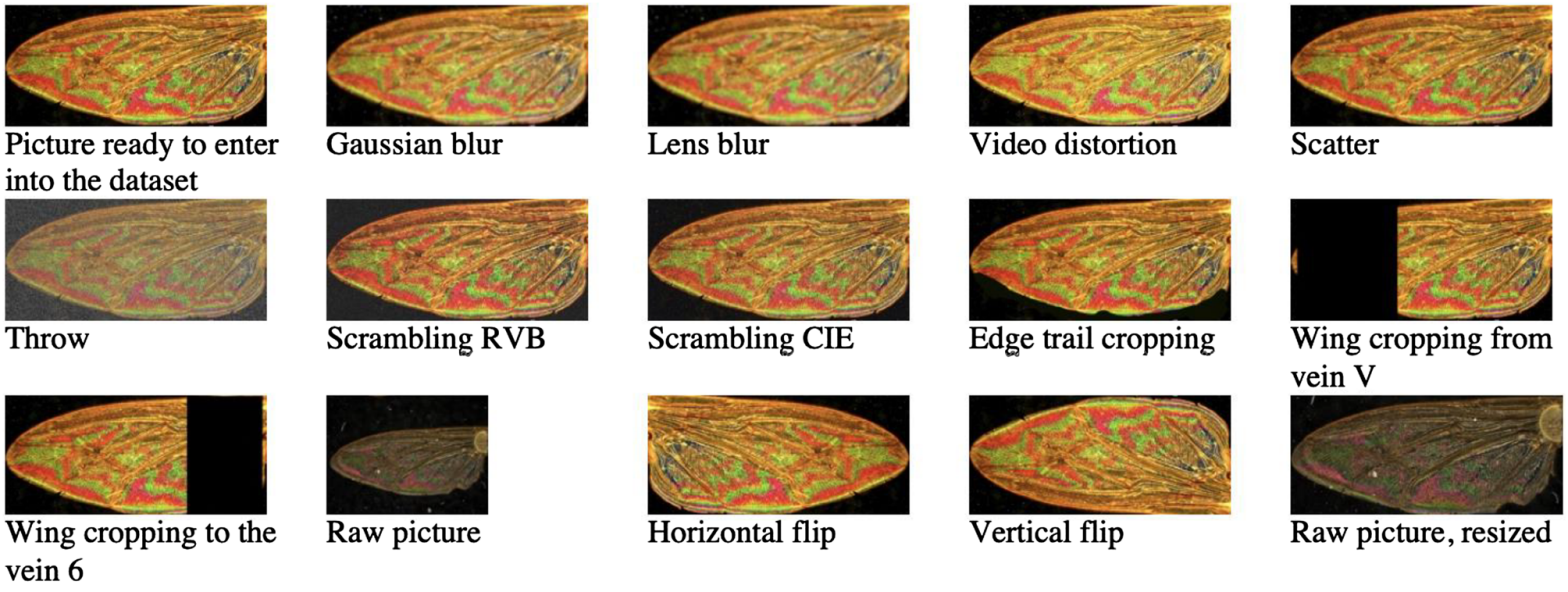
Examples of WIPs pictures (*G. pallidipes*) after manual or computer transformations.

We test the limits of the identification process according to image quality by performing a variety of numeric transformations, like blurring (Gaussian and lens blurring), distorting (video degradation), and adding interference (RGB and CIE degradation). Moreover, manual cropping of the picture, mimicking to some extent an event that can occur during *Glossina* trapping and/or processing of the sample, was performed (cropping the outer part of the wing at the vein V, or cropping the internal part of the wing at the vein VI, deleting the trailing edge). Examples of computer-aided and manually transformed pictures are given in Fig. 4. The set of images with their description is available on request to the corresponding authors.

Those transformed images were obtained using the Software GIMP version 2.10.30 (Available from: https://www.gimp.org)^21^. The following alterations were described in Table 2).

**Table 2:** Description of the transformation used for the evaluation of the algorithm robustness.

Transformation	Description	Simulated effect
Gaussian Blur	Image convolution with a 2d Gaussian filter with fixed standard-deviation	Foggy or out-of-focus lens
Lens Blur		Decentering of the lens
Video Distortion	Add scan lines in the image	Video display on a monitor
Spread	Swap each pixel with another randomly chosen pixel in proximity	(Light distortion)
Hurl	Pixels have a chance to get replaced by random color values	(Defective/Saturated pixels)
RGB Noise	Add a normally distributed noise	“Natural” looking noise
CIE Noise	Add normally distributed noise using the Lightness, Chroma, and Hue color model	
PWO	Partial wing occlusion (edge / external/internal)	Damaged wing
Flip	Horizontal/Vertical Flip	Variations in wing placement

## Results

### Wing Interferential Pattern according to Glossina wings genera, species, sex, and samples

**Figure 5:**
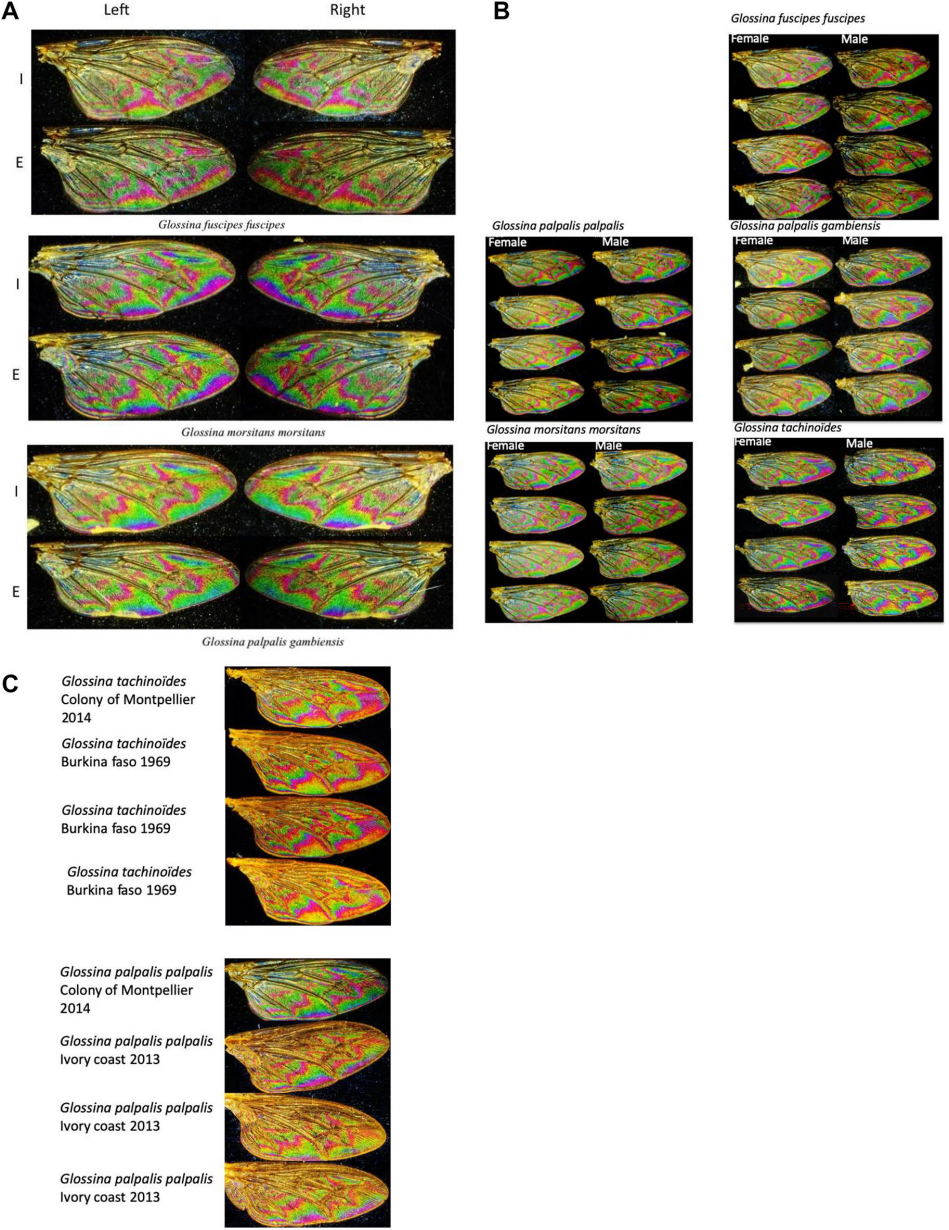
variability of WIPs generated on Glossina spp. according to (**A**) the wing orientation, top *G. f. fuscipes*, middle *G. m. morsitans* down *G. p. gambiensis*, (**B**) the samples, and (**C**) the preservation history. I, intrados and E, extrado.

To set up a protocol on which WIP can be acquired and used for Glossina species recognition, we performed experiments that allowed the visualization of WIPs under various conditions. Firstly, the conservation of the interferential pattern revealed on a wing of *Glossina* was analyzed according to the position of the radial symmetry (intrado/extrado) and axial symmetry (left and right). Following the process described in Fig. 1, pictures of *Glossina* specimens were taken. As exemplified in Fig. 5A demonstrating WIPs of *G. f. fuscipes*, *G. m. morsitans,* and *G. p. gambiensis*, no striking differences in the pattern of interferential colors were observed according to the wing position during image acquisition (intrado/extrado or right/left). Therefore, the positioning of the wing on the slide did not influence the WIP generated. To delineate the WIPs reproducibility, we further analyzed the stability of this phenotype on a large series of males and females specimen of various species (Fig. 5B). We noticed variation in the pattern of interferential color recorded on wings. This pattern was species-specific and presented a faint recurrent sexual dimorphism that must be further investigated. Finally, we investigated the stability of WIP according to the sampling date and the preservative mode. Discrete variations in the pattern of interferential light are recorded. Still, the overall pattern organization and its color composition remain similar, demonstrating the possibility of generating consistent interferential patterns from samples preserved in ethanol or air-dried for an extended period (Fig. 5C).

**Figure 6:**
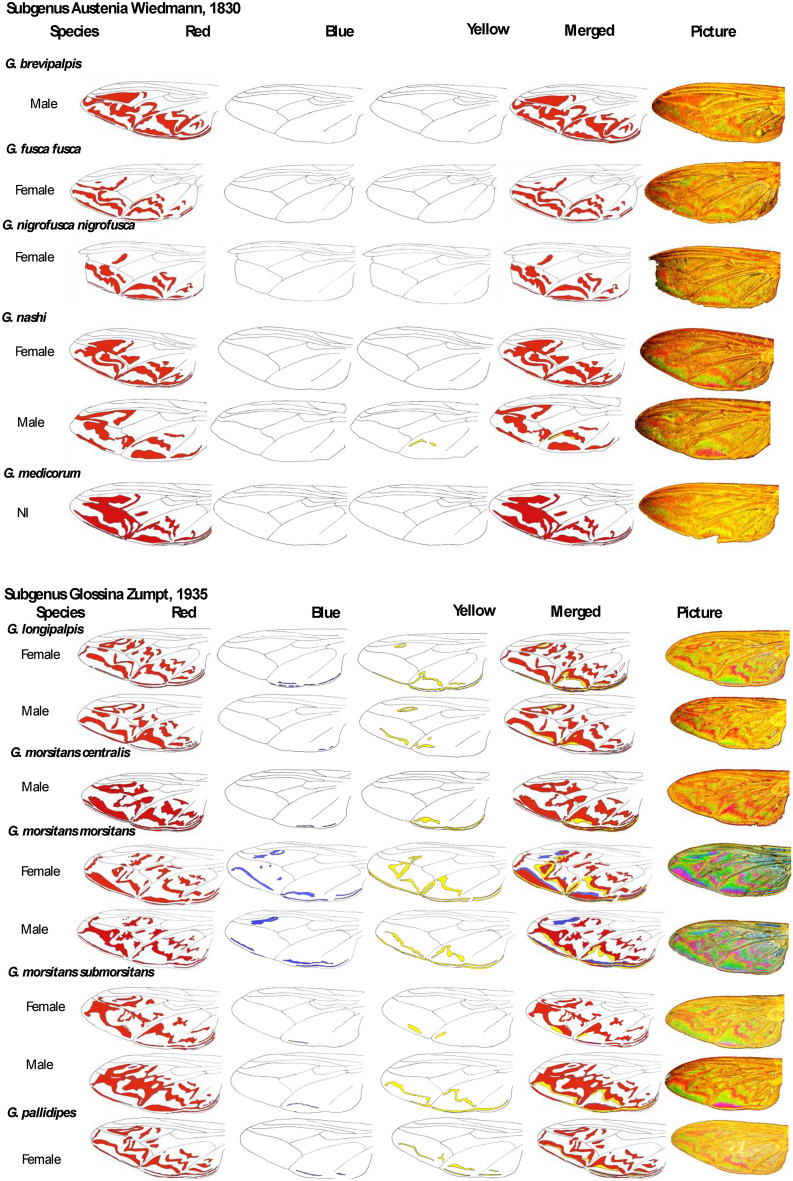

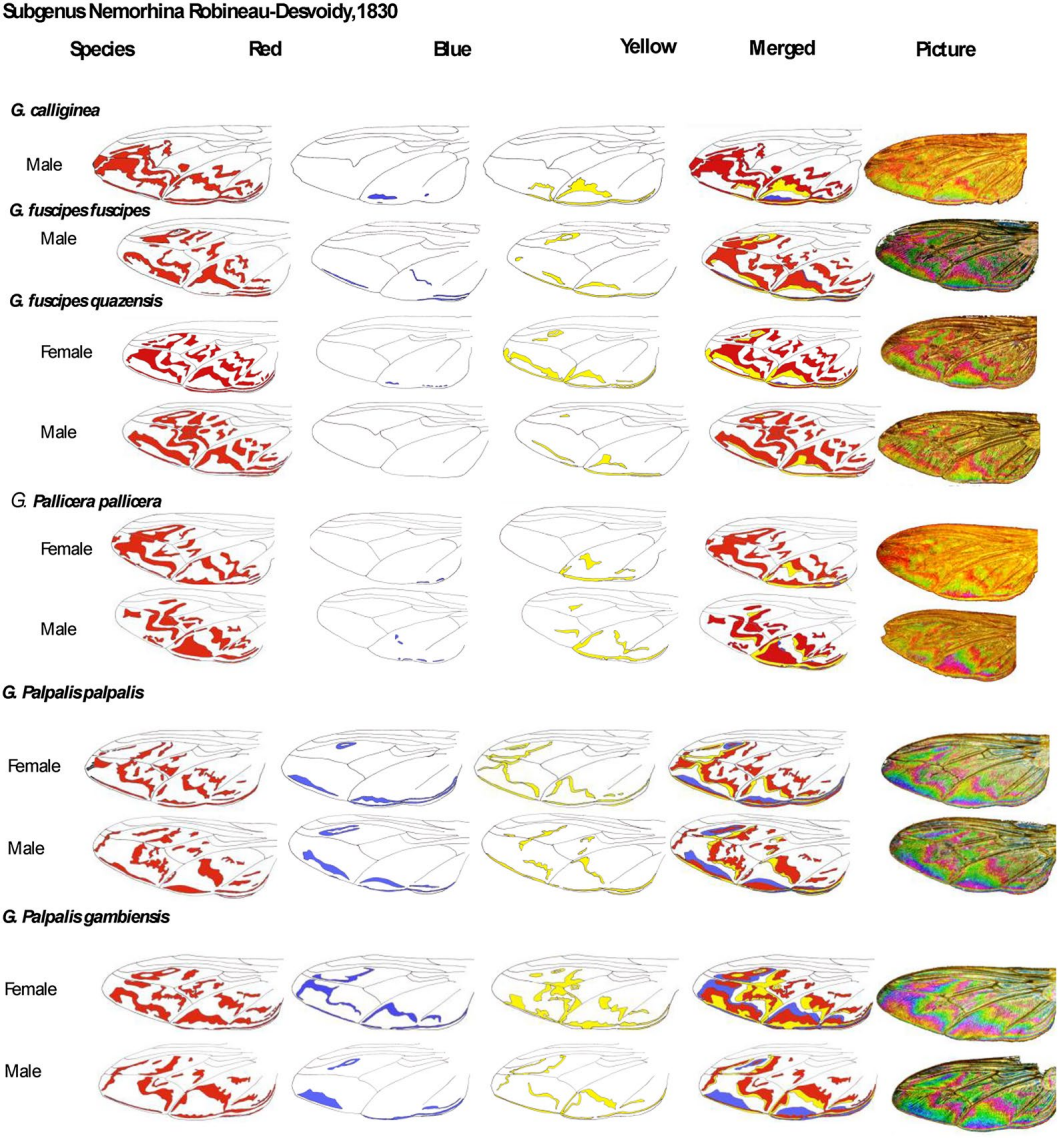
Selected *Glossina* spp pictorial key, deduced from Wing Interferential Pattern.

The wing color patterns were manually drawn from taken pictures (Fig. 6). This first pictorial was drawn using fresh field-caught specimens from Cameroun (P. Grebaut), or Ivory coast and previously used to perform geomorphometric analysis^28^, and for most specimens belonging to the IRD collection, see Table 1 for the characteristics of the specimen. For specimens of the collection, the identification was performed by an expert entomologist at the time of the flies’ capture (Table 1). The identification was performed for specimens from field traps, as previously reported^7^.

Strikingly, 4 prominent interferential colors are revealed on glossina wings, green, yellow, blue, and red. The pictorial key shows the red, blue, and yellow color patterns distribution according to the species and subspecies we gathered during the study (Fig. 6). The green color was not reported in the pictorial key because it represents the interferential background color and is thus figured as white in the pictorial key. The color diversity appeared to be lower for wings of *Glossina* species belonging to the *Austenia* subgenus, as compared to those belonging to *Nemorhina* or *Glossina* subgenera. Differences between species appeared related to the red pattern shape. *Glossina* species belonging to the *Nemorhina* and *Glossina* subgenera appeared to bear multicolor WIPs. The sexual dimorphism of this character was present in all samples, representatives of the species in which males and females were studied.

The interference pattern evidenced on the wing of the tsetse fly can help to set up an automatic identification system. A series of pictures of *Glossina* species and subspecies currently described, and the most important vectors of HAT and AAT, were taken. To test if such analysis can be considered as a fingerprinting approach for *Glossina* species identification, it is essential to discriminate most, if not all, *Glossina* species or subspecies currently known. As shown in Table 1, 23 out of 31 presently referenced *Glossina* species and subspecies were collected. They originate from the field, ARIM collection, or laboratory-reared *Glossina* flies.

### Training and classification

We explored training classifiers on the dataset alone and on a dataset where negative samples were added containing various non-*Glossina* insects as negative samples. Training the CNN (Convolutional Neural Network) on a combination of *Glossina* and non-*Glossina* images can improve the model to make correct predictions. The database was constructed by a total of 5516 pictures of dipteran insects WIPs in which 1766 pictures belonged to *Glossina* species. We deliberately crop and adjust all photos at the same dimension implying that (1) the size of wings cannot be used as a discriminative criterion for the classification process; (2) we cannot use landmarks to classify wings process. We primarily focused our analysis on *Glossina* species and subspecies documented as proven vectors for HAT and AAT, *i.e., G.p. palpalis, G. p. gambiensis*, *G. f. fuscipes*, *G. f. quazensis*, *G. f. martinii*, *G. m. morsitans*, *G. m. submorsitans*, *G. m. centralis*, *G. tachninoides*, *G. caligine*a, *G. swynertoni*, *G. pallidipides*, and *G. longipalpis*. Our database contains more than 80% of the *Glossina* species with medical or veterinary interest. Only *G. f. martinii* was absent in our database among Glossina species involved in Trypanosoma transmission. The dataset we constituted represents about 70% of species diversity as it contains WIPs pictures of 23 *Glossina* species and subspecies described.

Unfortunately, some were represented by only a few images, and for 9, no more than 15 specimens were used (Table 1). We then ascertained the accuracy of the classification process at various taxonomic levels of genus, species, and subspecies. The classifier demonstrated a high accuracy level of nearly 100% at the genus level, implying its competence in the classification/recognition of the *Glossina* genus (see Table 3A). In the next step, its performance in correctly assigning *Glossina* pictures at the species level was further challenged on complexes of species, *i.e*., *G. fuscipes*, *G. palpalis,* and *G. morsitans*. The classifier accuracy demonstrated incredible precision, with accuracy ranging from 90% for the *G. fuscipes* and *G. morsitans* complexes, to 100% for the *G. palpalis* ones (see Table 3B). We then further assessed the classification process at species and subspecies levels. At the time of the experiment, only 45% of *Glossina* species had entries with more than 8 pictures. Nevertheless, for almost all specimens tested, the classification accuracy also demonstrated a precision ranging from 33 to 100%. *Glossina palpalis palpalis* and *G. p. gambiensis* are primary vectors of HAT in West Africa. They can hybridize in the laboratory, but offspring males are sterile^23^. These two subspecies are challenging to identify, even if males show some morphological differences in the inferior clasper's terminal dilatation of their genitalia^24^. The deep learning methodology was somewhat highly accurate, with an accuracy of up to 97% (see Table 3C). Although the algorithm failed to identify 2 *Glossina* classes during the test, this can be explained, in part, by an extremely low number of WIPs pictures representative of the species in the test dataset (only 2 images for each class). Based on our dataset-splitting approach, we found these classes account for 8 images for training. This is a case of overfitting caused by insufficient training data, despite our self-imposed constraint of 10 total images per class. The results on the accuracy of Glossina classification are summarized in Table 3C.

**Table 3:**
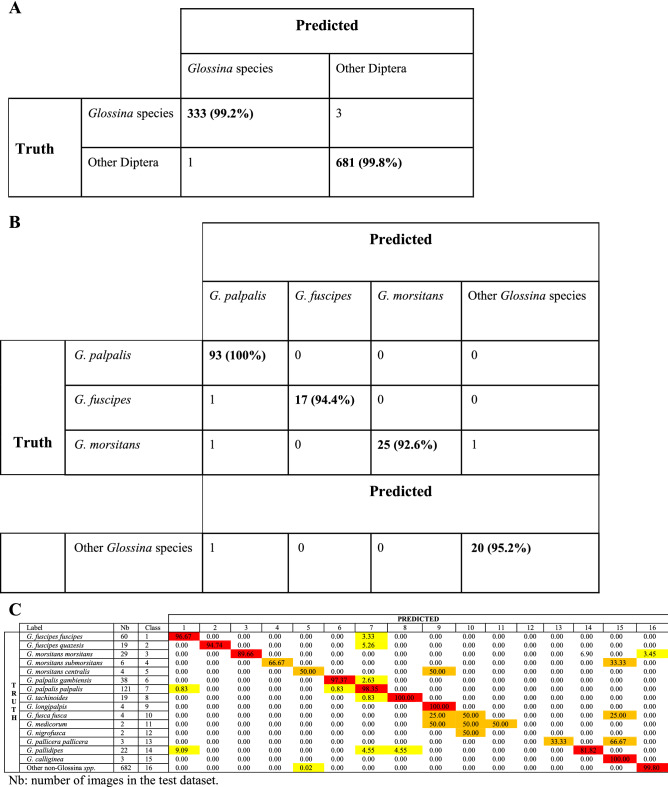
Results and confusion matrix for *Glossina* versus other genera (A) of the classification of specimens belonging to the palpalis, morsitans, and fuscipes complexes (B) and at the species and subspecies level (C).

Nb: number of images in the test dataset.

### Misclassified pictures

**Figure 7:**
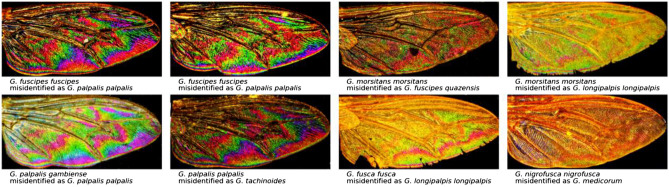
Misclassified picture; some examples from the images mistakenly predicted by the CNN model.

Inspecting a machine learning model for weak points would help identify underlying issues. This can be performed via a review of the miss-predicted images. This will get insights into what makes a photo hard to classify for the model. In Fig. 7, selected examples are presented. Deep learning models rely mainly on textures than on shapes. Therefore, a more extensive training set can avoid photo or sample quality pitfalls. To avoid confusing setups when taking photos; this can improve the accuracy of the automated classification. A guideline can be added to the application to advise participants to make high-qualified images of *Glossina* samples.

### Identification process examination following image transformation and cropping

Overall, a computer-aided and manual transformation of pictures is a tool to test the robustness of the identification process mimicking blur during image acquisition, image quality degradation, and integrity of samples’ wings. In addition, raw images of the acquisition process were tested for identification (Table 4). The modification was performed on chosen images from the training dataset (Table 4A) and the test dataset (Table 4B). In both cases, alterations of the image impact the identification accuracy. First, tsetse specimens insufficiently represented in the dataset failed to be identified (*Glossina fusca fusca* Walker, 1849). For most specimens, blurring (gaussian or lens) did not drastically modify the capacity of the trained model to identify specimens at the species level accurately. The video degradation affected the *Glossina* identification of some specimens. For *G. f. fuscipes*, *G. f. quazensis,* and *G. tachinoides*, the transformation did not impact the species identification, except in the case of throwing and scrambling (RVB) the image. In conclusion, with our trained model, the image alteration impeded the model's recognition capacity.

**Table 4:**
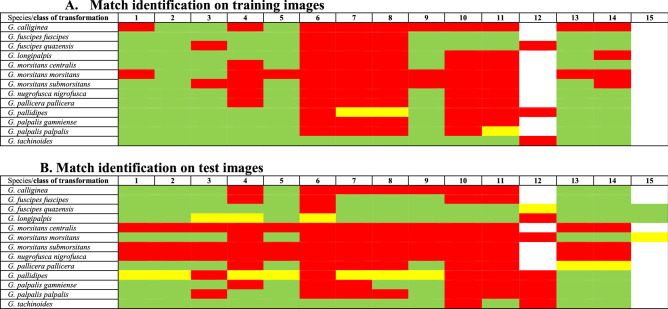
Identification accuracy on manually and computed transformed WIPs pictures, modified pictures taken in the training dataset (**A**) and the test dataset (**B**)

Legends: Green 100% match of identification, yellow identification below 100%, red no match of identification, white no samples submitted for identification. Transformation process using computer application (1 no transformation, 2 gaussian blurs, 3 lens blur, 4 video distortion, 5 scatter, 6 throws, 7 scrambling RVB, 8 scrambling CIE) or manual transformation (9 trailing edge cropping, 10 wings cropping from vein V, 11 wings cropping to the vein VI, 12 original picture without color saturation, contrast and resizing transformations, 13 horizontal flips, 14 vertical flips, 15 resized original picture).

## Discussion

The species identification of insects is crucial for an efficient survey of vectors involved in infectious diseases of public health importance. Taxonomists have access to sophisticated technologies relying on molecular identification via DNA barcoding^29,30^ and protein profiling (MALDI-TOF)^15^, or methodologies that capture images or sounds or even smell and taste of biological specimens. Nevertheless, most routine identification involves a small group of experts scattered around the world assessing diagnostic data qualitatively, commonly the size, shape, or texture of specimens or the presence or absence of certain features. The surveillance of arthropods of public health importance depends on non-expert or community participation and therefore focuses on a single or a limited number of vector species. For the survey, dichotomous keys have long been the primary tool for most taxonomic identification, although their use is limited by the expertise required. Both DNA barcoding and protein profiling via mass-spectrophotometry of proteins, as alternatives to morphology-based identification, are limited in their use because their expensiveness precludes their use outside research or emergency efforts. Therefore, in the context of climate change and the emergence of zoonotic infectious diseases, tools that can be translated into field applications are required to strengthen efforts in the survey of arthropods of medical and veterinary interest. This effort must involve automation of the identification process, avoiding the bottleneck of the need for expertise and simultaneously expanding the representatives of the arthropods of medical and veterinary interest surveyed. This would be crucial for vector-borne transmitted Neglected Tropical Diseases (Leishmaniasis, Trypanosomiasis, Dengue, Zika…), where specialists are needed to train entomologists capable of distinguishing between vector species and evaluating their dynamics. It is a labor-intensive and time-consuming mission requiring financial and human resources devoted to active vector control. Hopes were high among researchers and funding bodies that DNA barcoding, by which a species is recognized according to a marker in its mitochondrial genome, will increase the accuracy of identifications and ease bottlenecks resulting from a shortage of trained and experienced taxonomists^31^. Nevertheless, stakeholders have overlooked the greater promise of machine learning to transform taxonomy and the identification of natural objects in general by focusing on DNA barcoding. Digital Automated Identification SYstem (DAISY), developed by Mark. A. O’Neill, in 2010, is accessible for classifying objects into 2 to 30 categories^31^. These systems deliver faster, more accurate, and more consistent semi or fully-automated identification than any human taxonomist. For instance, a group of entomologists at the Natural history museum in London have used the Digital Automated Identification System (DAISY) to identify 15 species of parasitic wasps via digital images of wings, with 100% accuracy, each identification taking a few seconds^32^. The need for community participation in blood-sucking invasive species identification, for example, *Aedes (Ochloretatus) albopictus*, Skuse 1895 has pushed deep learning methodology in the entomological survey field. An increasing number of studies are published, focusing solely on this invasive species with identification challenges on imago^33–36^ or larval stage^37^. In addition, the design of traps with embedded systems for counting trapped insects opens up possibilities for real-time surveillance of insect density, a crucial parameter in the survey of insect vectors of medical or veterinary interest^38^. All these studies do not focus on and do not take advantage of the interferential colors generated at the surfaces of an insect wing, even if more and more studies on their taxonomic interest for insects are published^18,19,39–41^. This is the first study suggesting a combination of WIPs detection at the surface of insect wings and deep learning for insects’ identification^42^.

Dichotomous keys for tsetse identification, based on morphological discriminant characteristics, are available as paper^43^, or as software^7^. Till now, Glossina identification has been based almost exclusively on the presence of morphological discriminative criteria of species or subspecies since no information is available on DNA barcoding for tsetse flies (http://v4.boldsystems.org/index.php), and two reports relate identification using protein profiling by mass spectrometry (MALDI-TOF)^9,10^. Landmark and outline morphometric of the central cell of the wing are considered important taxonomic significance criteria for tsetse flies^44^, which disclose accuracy in the identification varying between 66 to 77%^45^, depending on the taxonomic level of the specimen and restricted diversity of species considered by these studies. Such scarcity of alternative ways to identify tsetse fly would severely impact the surveys on these vector flies required for the future of the elimination program of sleeping sickness as a public health concern and in the imprudent of food security in Africa. Our results point out that species and subspecies delineation will be amenable for tsetse flies. Nevertheless, even if our dataset is rich in terms of species diversity, serious efforts must be undertaken to supplement it with properly qualified pictures of most, if not all, *Glossina* species and subspecies currently described. Another aspect of the identification process using image acquisition would be its potential to be used on smartphones.

The advantages of our computer-aided methodology for species and subspecies recognition of Glossina flies are summarized compared with the other methodology available in Table 5 with respect to five criteria.

**Table 5:** Synthetic view of advantages and limitations of some identification methodologies for medically important arthropods.

Methods	Conservation procedure for identification	Technical cost*	Computational cost	Effort	Sample destruction	Precision (Sp/Ssp)	Available for Glossina spp
DNA Barcoding	−20 °C	High	Low	High	Yes	Ssp	No
Protein profiling	4 °C at best	High	Low	Low	Yes/part	Sp	3 Glossina sp
Dichotomous keys	RT	High	High	High	No/yes$	Ssp	Yes
WIPs (supervised)	RT	Low	None	Low	No	Ssp	Yes
WIPs + Deep Learning (ours)	RT	Low	Medium	Medium	No	Ssp	Yes

*The technical cost includes sample preparation (DNA, protein extraction, slides, etc.) and expenses linked to the need for a skilled professional. $ Samples destruction can be necessary for some species of medically important insects like *Psychodidae,* sp species, Ssp subspecies.

The application of Deep learning leads to robust results in terms of classification performance. Therefore, it is worth evaluating, even qualitatively, if the proposed approach could be usable in real-life scenarios regarding several important criteria: cost, computational resources, analyzing time, damaged samples, and the taxonomic level of the classification. Considering the mentioned criteria, our proposed architecture ends up with a good compromise compared to other methodologies and the ability to classify up to the subspecies taxonomic level. This architecture was thought to be used on portable devices.

Future development and technical implementation of this methodology include the following aspects:

- To strengthen the database in terms of species diversity, the use of GANs (Generative adversarial network) will allow filling up the database with new species, even with a low number of representatives. This DL approach makes it possible to generate original samples with characteristics learned on the initial database.
- A key point of DL approaches is the loss function used during the training process for updating the architecture weights. By default, the cross-entropy function was used here, but other types of loss with a better fit to the kind of data would improve the performance, and these must be investigated.

Second, from an application point of view:

- This work was carried out on tsetse. It can be extended to other insect families have to be carried to check to what extent such approaches can be used as a generic one for dipteran insect identification.
- Implementing a Mobile app. and SaaS platform would offer a complete service for both in-the-field and remotely localized computers, with an internet connection.

Finally, from a long-term perspective, it is promising to consider how to go towards a WIP-based method for living insect identification with no need for captures and preparation.

## Data Availability

Database of Glossina species WIPS (1766 picture) is available on request to Denis SERENO (denis.sereno@ird.fr).
